# Need for Hepatitis B Vaccination in Medical Students

**DOI:** 10.31729/jnma.8189

**Published:** 2023-06-30

**Authors:** Niranjan Chapagain, Sanskriti Chapagain

**Affiliations:** 1 Nepalese Army Institute of Health Sciences, Sanobharyang, Kathmandu, Nepal; 2 Devdaha Medical College and Research Institute, Bhaluhi, Rupandehi, Nepal

**Keywords:** *autopsy*, *hepatitis B vaccine*, *medical students*

## Abstract

Hepatitis B is a global acute and chronic life-threatening liver infection caused by the hepatitis B virus. During cadaveric dissection, clinical rotations, and autopsy posting, medical students get exposed to patients' blood and body fluids, which increases the risk of hepatitis B infection. Hepatitis B can be prevented by vaccines that are safe, easily available, and effective, however many medical students are still unvaccinated. This results in the need for attention to prevent early exposure and provide vaccination at regular intervals during clinical training and professional practice.

## INTRODUCTION

Hepatitis B virus (HBV) infection is a major global health problem that can cause chronic liver disease and increase the risk of cirrhosis and liver cancer.^1^ Medical students are at significant risk of unintentional exposure to HBV infection due to their lack of experience and close contact with patients' potentially infected bodily fluids. A study conducted on 210 medical students during their clinical rotations revealed that 90 of them reported experiencing at least one injury, out of which 4 students had been exposed to cases positive for HBV.^2^ Patients may also be at risk from HBV-infected healthcare workers, as there is a known possibility of HBV transmission from treating physicians. Hepatitis B is a vaccine-preventable disease with a vaccination efficacy of about 90-95%, however, vaccination practices are not well received among medical students.^3^

## MEDICAL STUDENTS AND HBV INFECTION

Medical students build their clinical knowledge on the ground of their preclinical years. While practising cadaveric dissection in the anatomy lab, we noticed frequent mishandling of scalpels which led to accidental injuries, and hazardous splashes to the eyes, nose, and mouth in many of our colleagues. A study shows that a person who died of Hepatitis B is still contagious when it arrived in the anatomy department as a corpse.^4^ This depicts the risk of contracting HBV infection during cadaveric dissection in medical students, as most of us are unvaccinated.^2^

In clinical years, patient interaction while bedside history taking and clinical examination facilitates an important learning resource. During this process, we also perform various procedures like drawing blood, opening IV lines, and suturing minor wounds, where we tend to miss out on basic preventive measures like proper use of gloves and hand washing before and after the procedure, which is likely to expose us to HBV infection. If one sustains a needle stick and the patient is an infected one, the risk of transmission of HBV per exposure is 37-62%.^2^ HBV has been shown to survive in dried blood on environmental surfaces like table tops, blades, and blood stains for at least a week without losing its infectivity. Moreover, direct or indirect blood or body fluid exposures that inoculate HBV into cutaneous scratches, abrasions, burns, other lesions, or mucosal surfaces can cause HBV infections to occur in medical students even without a history of percutaneous injury.^5^

Transmission of the virus may also occur with exposure to infected blood and body fluids, such as menstrual, vaginal, and seminal fluids.^1^ In our gynaecology and obstetrics posting, we might come in contact with vaginal secretions and body fluids. As part of our autopsy posting, we had to collect tissue and fluid samples for postmortem examination along with examination of the internal organs for any injuries or diseases that may have contributed to or caused the death. Autopsy specimens are potential sources of HBV infection and protection against HBV infection is extensively provided by vaccinations and targeted immunoglobulins.^6^

## PREVENTION AND VACCINATION

Medical students can reduce their risk of exposure to blood and body fluids through the use of standard precautions, which include hand hygiene, the use of personal protective equipment (PPE), respiratory hygiene, double gloving, sharps safety, safe injection practices, sterile instruments and devices, and clean and disinfected environmental surfaces.^7^

Vaccination against HBV infection is mandatory for medical students in some countries like Belgium and France, while it is recommended in countries like UK and Spain.^8^ Although the primary method of preventing Hepatitis B infection is vaccination, the government and institutions lack programs to vaccinate medical students. Most of us born before 2002 AD were unvaccinated as newborns since no vaccination program was offered then.^2^ Those who have already received vaccinations are still prone to infections as the efficacy of vaccines decreases over time.^3^ Unvaccinated individuals not only put themselves but also their friends and family members at risk of getting the infection. Therefore, every medical student must be motivated to complete all three doses of hepatitis B vaccination early after the start of their career to avoid infection and development of carrier status.^8^

## WAY FORWARD

The level of exposure to blood and other body fluids among medical students is note-worthy, which increases the risk of HBV infection, which, fortunately, is a vaccine-preventable disease. But the incidence of vaccination among medical students is very low. Therefore, attention must be given to preventing exposure among students early in medical school, and they should undergo vaccination at regular intervals during their clinical training and professional practice.
